# Depressive symptoms in workers with subsistence jobs in the pandemic context, according to working and extra-working conditions, Medellín, Colombia 2021

**DOI:** 10.47626/1679-4435-2024-1276

**Published:** 2025-07-13

**Authors:** María Osley Garzón-Duque, Fabio León Rodriguez-Ospina, Estefanía Uribe Vallejo, Carolina Jerez Vélez, Tatiana Elizabeth Reina-Jojoa, Jennifer Alejandra Giraldo Ciro, Marcela Vargas Gómez

**Affiliations:** 1 Facultad de Medicina, Universidad CES, Medellín, Antioquia, Colombia; 2 Facultad Nacional de Salud Pública, Universidad de Antioquia, Medellín, Antioquia, Colombia

**Keywords:** informal sector, working conditions, depression, mental health, COVID-19, sector informal, condiciones de trabajo, depresión, salud mental, COVID-19

## Abstract

**Introduction:**

Although mental health is a topic of interest in public health, there is still little evidence on depressive symptoms among informal workers in times of pandemic.

**Objectives:**

To identify the prevalence of depressive symptoms and their relationship with working and socioeconomic conditions among informal workers in the context of the pandemic, Medellin, Colombia, 2021.

**Methods:**

Cross-sectional study using primary data collected from 656 workers, after obtaining informed consent. A pilot test was conducted, and selection and information biases were controlled. Exploratory, bivariate, and multivariate analyses were performed using logistic regression.

**Results:**

A higher proportion of participants were men; 74% were aged ≥ 45 years, 85% were heads of household and experienced economic, labor and health difficulties during the isolation period. A prevalence of moderate/severe depressive symptoms of 4.3% was identified. These were associated (p < 0.05) with age (30 and 44 years old), belonging to a single-parent family, living in an urban area, consuming alcohol , lacking work authorization, not having received economic support during mandatory isolation, and having received economic support from their labor association. Higher levels of depressive symptoms were explained (p < 0.05) by age (30 and 44 years old), belonging to a blended or single-parent family, consuming alcohol, and not having received economic support during quarantine.

**Conclusions:**

The conditions that explain a higher prevalence of moderate/severe depressive symptoms in this population can be addressed in future emergencies through joint actions by the State, the families, and workers.

## Abstract (pt)

**Introducción:**

Aunque la salud mental es un tema de interés en salud pública, aún existe poca evidencia sobre la sintomatología depresiva en trabajadores informales en época de pandemia.

**Objetivos:**

Identificar la prevalencia de sintomatología depresiva, y su relación con las condiciones laborales y socioeconómicas en trabajadores informales en el contexto de la pandemia, Medellín, Colombia, 2021.

**Métodos:**

Estudio transversal utilizando datos primarios recolectados de 656 trabajadores, previa obtención del consentimiento informado. Se realizó una prueba piloto y se controlaron los sesgos de selección e información. Se realizaron análisis exploratorios, bivariados y multivariados con regresión logística.

**Resultados:**

Se observó mayor proporción de hombres; el 74% tenía ≥ 45 años, el 85% era cabeza de hogar, con dificultades económicas, laborales y de salud durante el aislamiento. Se identificó una prevalencia de sintomatología depresiva moderada/severa del 4,3%. Esta se asoció (p < 0,05) con la edad (tener entre 30 y 44 años), pertenecer a una familia monoparental, residir en zona urbana, consumir licor, no tener permiso para trabajar, no haber recibido apoyo económico durante el aislamiento obligatorio, y haber recibido apoyo económico de su asociación laboral. Una mayor sintomatología depresiva se explicó (p < 0,05) por la edad (tener entre 30 y 44 años), pertenecer a una familia compuesta o monoparental, consumir licor, y no haber recibido apoyo económico durante la cuarentena.

**Conclusiones:**

Las condiciones que explican una mayor prevalencia de sintomatología depresiva moderada/severa en esta población pueden ser abordadas en futuras emergencias mediante acciones conjuntas del Estado, las familias y el trabajador.

## INTRODUCTION

Informality is a socioeconomic phenomenon characterized by noncompliance with labor, tax, and social security regulations.^1^ Working conditions within this context foster a state of vulnerability that can lead to serious implications for workers’ health and quality of life. This issue is particularly concerning given that approximately two billion individuals worldwide (61% of the labor force) are employed informally^2^ and, in Latin America and the Caribbean, this includes at least 140 million people, representing about 50% of the region’s workforce.^3^

In Colombia, the National Administrative Department of Statistics (Departamento Administrativo Nacional de Estadística, DANE), through the Great Integrated Household Survey (Gran Encuesta Integrada de Hogares, GEIH) reported an informal employment rate of 49.2% in 23 cities and metropolitan areas between December 2019 and February 2020.^4^ This condition reflects exposure to inadequate working conditions, uncertain and irregular income, long working hours, and a lack of rights to collective bargaining and representation.^5^

In the context prior to the COVID-19 pandemic, data indicated a higher prevalence of depressive symptoms among workers engaged in subsistence jobs, with several associated risk factors identified in this population, such older age, chronic health conditions, limited access to healthcare, and use of psychoactive substances.^6^ By 2019, in Medellín, Colombia, various occupational, socioeconomic, and environmental conditions, as well as certain habits, had been linked to a higher prevalence of depressive symptoms among this group of workers. These included working more than 8 hours a day (47%), perceived exposure to noise pollution (29%), and food insecurity (36%), among others.^7^

However, the pandemic may have affected the working population in different ways; some sectors of the economy, such as the informal sector, were more vulnerable than others. The precarious conditions and absence of adequate social protection to cope with the suspension of economic activity may have triggered major psychological changes, such as high levels of stress, anxiety, and depression.^8^ In this context, a study conducted in La Paz, Bolivia, on the psychosocial conditions of informal workers during the pandemic, reported the following results: 16% experienced depression, 24% anxiety, 26% stress, and 27% psychological impact. In addition, psychological symptoms were more prevalent among workers who did not share household chores.^9^ However, there is still limited documentation on the conditions faced by subsistence workers operating on the streets and sidewalks of Colombian cities like Medellín.

Considering the reasons outlined above and the emergence of the pandemic, this study aimed to advance the production of evidence concerning the mental health of the study population. Specifically, it sought to identify the prevalence of depressive symptoms and their relationship with sociodemographic, economic, social support factors, as well as working, workplace environmental, and mandatory isolation conditions, in order to determine possible variations in these characteristics. The findings may inform the planning of actions to improve living and health conditions and to better anticipate and respond to future pandemics, emergencies, or disasters.

## METHODS

### DESIGN AND POPULATION

**Figure 1.**
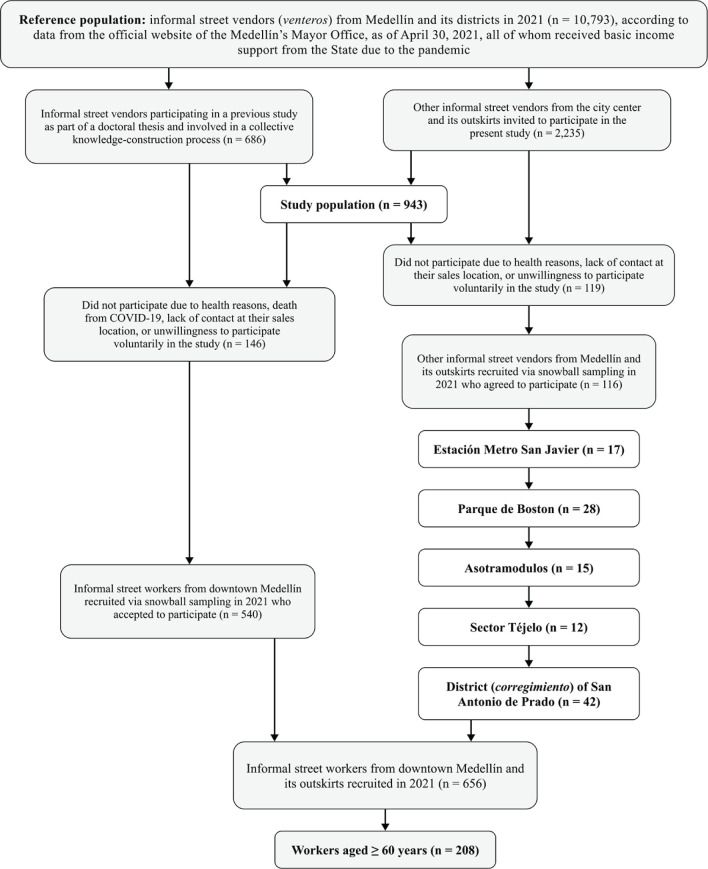
Diagram of population selection for the present study on depressive symptoms among subsistence workers.

A cross-sectional analytical study was carried out based on primary data collected through assisted surveys conducted between March and November 2021 among 656 informal street vendors (*venteros*) from Medellín and the district (*corregimiento*) of San Antonio de Prado, selected via snowball sampling (Figure 1). This article stems from the main project titled “Living, working, and health conditions among informal street vendors in Medellín during and after the COVID-19 pandemic (2021–2022),” approved in January 2021 by the Institutional Human Research Ethics Committee of Universidad CES under record 156, project 965, and classified as posing minimal risk. Informed consent was obtained from all participants.

Contact with workers was made through their leaders by the principal investigator and a co-investigator during trade union meetings and assemblies.

### INCLUSION CRITERIA

Participants were workers aged ≥ 18 years, with at least 3 years of occupational experience, who were informed about the study and its procedures, expressed their voluntary willingness to participate, and signed the informed form prior to data collection. No participants were excluded based on the following exclusion criteria: being under the influence of psychoactive substances, failing to complete ≥ 50% of the survey, requesting to move to a different location to complete the survey, or failing to sign the informed consent form.

### VARIABLES

#### Dependent variable

Perception of depressive symptoms was assessed using the Zung Self-Rating Depression Scale (SDS),^10^ a 20-item questionnaire with total scores ranging from 20 to 80 points^10^ and classified as follows: absence of depression (20 to 35 points); subclinical depression (36 to 51 points); moderate-to-severe depression (52 to 67 points); and severe depression (68 to 80 points). For bivariate and multivariate analyses, scores were recategorized into two groups: 1. absence depression or subclinical depression (20 to 51 points); 2. moderate-to-severe depression (52 to 67 points). No participant scored in the severe depression category.

#### Independent variables

Sociodemographic, behavioral, family dysfunction, and food insecurity variables were collected, as well as economic conditions, support received, working and environmental conditions, and aspects related to mandatory isolation. These included: 1) sociodemographic, behavioral, family dysfunction, and food insecurity variables: sex, age, area of residence within the municipality of origin, family type, cohabitation with other families, marital status, educational level, household stratum, food insecurity, family dysfunction, use of alcohol and tobacco; 2) economic variables: monthly income and whether the person was the head of household; 3) support during the pandemic: whether any support was received from public or private entities, peers, or associations, and type of support received; 4) work and environmental conditions: job stability before and during the pandemic, work authorization, whether this authorization provided emotional support, occupational experience, job satisfaction, number of workdays per week, working hours per day, and discomfort due to chemical exposure and noise at the workplace; and 5) mandatory isolation: whether the person self-isolated, duration of isolation, whom they lived with during the quarantine, and whether the family unit was disrupted during lockdown.

Prior to data collection, the research team was standardized, and a pilot study was conducted with seven workers. Selection bias was controlled through adherence to inclusion criteria and the use of snowball sampling to recruit participants. Information bias was addressed at multiple levels: in the instrument (validated in both form and content by the workers themselves, their leaders, and experts on the subject); in the interviewer (through standardization and pilot testing); and in the participants (via sensitization and explanation of the project, its objectives, scope, benefits, and limitations). The analyses were adjusted for potential confounders: sex, age, and occupational experience.

### DATA ANALYSIS

#### Descriptive analyses

Frequency distributions and percentages were calculated for all the variables.

Bivariate analysis – Associations between depressive symptoms and the independent variables were assessed using the chi-square test. The strength of the association between depressive symptoms and the independent variables was estimated using prevalence ratios (PR) and their corresponding 95%CI.

Multivariate analysis – Explanatory binary logistic regression was performed, including independent variables with a p-value ≤ 0.10. Descriptive and bivariate analysis were conducted using Epidat 3.1, while multivariate analysis was performed with SPSS version 26, licensed by the Universidad de Antioquia.

## RESULTS

### SOCIODEMOGRAPHIC AND ECONOMIC CHARACTERISTICS

Overall, 41.9% of participants were men, and most participants were aged from 45 to 59 years, with a predominance of women among those younger than 59 years of age. Most workers (53.3%) lived in urban areas, and 70.2% in households belonging to the very low and low socioeconomic strata (Table 1).

**Table 1:** Descriptive statistics of sociodemographic and economic conditions, social support, and mandatory pandemic isolation, according to workers’ biological sex, Medellín, Colombia, 2021

Variable	Sex				Total	
	Men		Women			
	n	%	n	%	N	%
Age in years						
18-29	10	2.7	14	4.9	24	3.7
30-44	62	16.8	80	28.0	142	21.6
45-59	155	41.9	127	44.4	282	43.0
≥ 60	143	38.6	65	22.7	208	31.7
Area of residence within the municipality of origin (n = 651)						
Urban	187	51.0	173	61.0	360	55.3
Rural	180	49.0	111	39.0	291	44.7
Head of household						
Yes	301	81.6	257	89.9	558	85.2
No	68	18.4	29	10.1	97	14.8
Family type						
Nuclear	207	55.9	153	53.5	360	54.9
Extended	0	0.0	2	0.7	2	0.3
Blended	49	13.2	75	26.2	124	18.9
One-person	84	22.7	26	9.1	110	16.8
One-parent	2	0.5	6	2.1	8	1.2
Restructured.	28	7.6	24	8.4	52	7.9
Cohabitation with other families						
Yes	59	15.9	40	14.0	99	15.1
No	311	84.1	246	84.0	557	84.9
Marital status						
Without a partner	156	42.2	214	74.8	370	56.4
With a partner	214	57.8	72	25.2	286	43.6
Educational level in years						
0-5	243	65.7	155	54.2	398	60.7
6-10	59	16.0	60	21.0	119	9.1
> 10	68	18.4	71	24.8	139	21.2
Monthly income in Colombian pesos						
30.000-150.000	20	5.4	26	9.1	46	7.0
151.000-300.000	50	13.5	78	27.3	128	19.5
301.000-500.000	71	19.2	62	21.7	133	20.3
501.000-800.000	130	35.1	72	25.2	202	30.8
801.000-1.000.000	73	19.7	39	13.6	112	32.3
> 1.000.000	26	7.0	9	3.1	35	5.3
Household socioeconomic stratum						
Zero	8	2.2	6	2.1	14	2.1
Very low	85	23.0	93	32.5	178	27.1
Low	166	44.9	117	41.0	283	43.1
Lower-middle	95	25.7	56	19.6	151	23.0
Middle	15	4.1	12	4.2	27	4.1
Habits, emotional symptoms, and family dysfunction						
Smoking						
Yes	82	22.2	43	15.1	125	19.1
No	288	77.8	242	84.9	530	80.9
Alcohol consumption						
Yes	102	27.6	35	12.2	137	21.0
No	267	72.4	251	87.8	518	79.0
Moderate/severe depressive symptoms						
Yes	16	4.3	12	4.2	28	4.3
No	354	95.7	274	95.8.	628	95.7
Family dysfunction						
Severe	89	24.0	82	28.7	171	26.1
Moderate	79	21.3	58	20.3	137	20.9
Mild	74	20.0	66	23.0	140	21.3
Absent	128	34.6	80	28.0	208	31.7
Self-isolated during the pandemic						
Yes	347	93.8	280	97.9	627	95.6
No	23	6.2	6	2.1	29	4.4
Duration of self-isolation in weeks (n = 628)						
Did not self-isolate	0	0.0	1	0.4	1	0.2
< 1	0	0.0	0	0.0	0	0.0
1	0	0.0	1	0.4	1	0.2
2	6	1.7	7	2.5	13	2.1
3-4	27	7.8	15	5.3	42	6.7
5-8	29	8.4	11	3.9	40	6.3
9-12	43	12.4	28	9.9	71	11.3
> 12	242	69.7	218	77.6	460	73.2
Whom they lived with during the quarantine						
Family at their household	301	81.4	228	79.7	529	80.6
Relatives at another household	25	6.8	40	13.9	65	10.0
Relatives or friends	4	1.1	2	0.7	6	0.9
Peer vendors	5	1.3	1	0.3	6	0.9
Other	35	9.4	15	5.2	50	7.6
Family unit disrupted during quarantine						
Yes	22	5.9	27	9.4	49	7.5
No	347	94.1	259	90.6	606	92.5
Received support from public/private entities (n = 653)						
Yes	207	55.9	169	59.1	375	57.4
No	163	44.1	114	40.9	277	42.6
Type of support received from private entities (n = 377)						
Emotional	0	0.0	0	0.0	0	0.0
Financial	7	3.4	3	1.8	10	2.7
Physical	0	0.0	0	0.0	0	0.0
Food aid	201	96.6	166	98.2	367	97.3
Psychosocial	0	0.0	0	0.0	0	0.0
Received support from peer vendors (n = 654)						
Yes	19	5.1	12	4.1	31	4.7
No	350	94.9	273	95.9	623	95.3
Received support from their labor association						
Yes	185	50.0	157	54.9	342	52.2
No	185	50.0	128	45.1	313	47.8
Type of support received from the association (n = 341)						
Emotional	3	1.6	0	0.0	3	0.9
Financial	2	1.1	2	1.3	4	1.2
Physical	0	0.0	0	0.0	0	0.0
Food aid	179	97.3	155	98.7	334	97.9
Psychosocial or other	0	0.0	0	0.0	0	0.0
Peer vendors were sources of support in times of hardship or crisis						
Yes	195	52.7	149	52.3	344	52.5
No	175	47.3	136	47.7	311	47.5
Their job provided emotional support (n = 655)						
Yes	368	99.5	280	98.2	648	98.9
No	2	0.5	5	1.8	7	1.1
Membership in a labor association offered social support						
Yes	313	84.6	241	84.2	554	84.5
No	57	15.4	45	15.8	102	15.5

In terms of family type, 54.9% of participants had a nuclear family, 18.9% had a blended family, and 16.8% lived alone. A total of 60.7% of workers had from 0 to 5 years of education, with a similar distribution across sexes. Of note, 81.6 % of men and 89.9 % of women were the main income earners in their household. Moreover, 42.2% of men and 74.8% of women did not have a partner (Table 1).

#### Habits of the surveyed working population

In relation to lifestyle habits, 22.2% of men and 15.1% of women reported smoking. Alcohol consumption was reported by 27.6 % of men and 12.2 % of women (Table 1).

### CONDITIONS REGARDING ISOLATION AND SUPPORT RECEIVED DURING THE PANDEMIC

A total of 95.6% of workers underwent mandatory quarantine and, regarding its duration, 69.7% of men and 77.6% of women remained in isolation for > 12 weeks. Most workers (80.6%) quarantined with their family in their own residence, while 6.8% of men and 13.9% of women isolated with relatives in another household. Family unit was disrupted in 5.9% of men and 9.4% of women (Table 1).

During the pandemic, 57.4% of workers received support from public or private entities. In the case of private entities, support was provided primarily in the form of food assistance (97.3%) and, to a lesser extent, financial aid (2.7%). While only 5.1% of men and 4.1% of women received support from their peer vendors during the pandemic, 52.5% identified them as potential sources of support in times of hardship or crisis. A total of 98.9% of participants reported that their job provided emotional support, and 84.5% stated that membership in a labor association offered social support (Table 1).

### WORKING CONDITIONS

Assessment of job stability revealed that, before the pandemic, 14.2% of participants rated it as very good, 65.2% as good, and 19.2% as fair. During the pandemic, these figures shifted to 0.6%, 11.9%, and 66.3%, respectively. Although only half of the workers had work authorization, 97.9% perceived it as a form of social support. Moreover, 98.3% worked ≥ 4 días a week, and 79.1% worked ≥ 9 hours daily. A total of 59% had ≥ 21 years of occupational experience, with men showing a higher prevalence among those with > 30 years of occupational experience. Finally, 93.4% felt satisfied with their job (Table 2).

**Table 2:** Working conditions according to participants’ biological sex, Medellín-Colombia, 2021

Variable	Sexo				Total	
	Hombre		Mujer			
	n	%	n	%	N	%
Job stability before the pandemic						
Very good	61	16.5	32	11.2	93	14.2
Good	238	64.3	190	66.4	428	65.2
Fair	67	18.1	59	20.7	126	19.2
Poor	4	1.1	4	1.4	8	1.2
Very poor	0	0.0	0	0.0	0	0.0
Do not know	0	0.0	1	0.3	1	0.2
Work authorization						
Yes	200	54.0	133	46.5	333	50.7
No	170	46.0	153	53.5	323	49.3
Use of chemical substances at their workplace that cause discomfort						
Yes	15	4.1	10	3.5	25	3.8
No	355	95.9	276	96.5	631	96.2
Number of workdays a week						
1-3	1	0.3	10	3.5	11	1.7
4-6	211	57.0	164	57.3	375	57.2
7	158	42.7	112	39.1	270	41.1
Food security according to ELCSA						
Moderate/severe insecurity	142	38.4	147	51.4	289	44.1
Food security/mild insecurity	228	61.6	139	48.6	367	55.9
Feel satisfied with their job (n = 654)						
Yes	346	93.5	268	94.0	614	93.9
No	23	6.5	17	6.0	40	6.1
Air quality of their workplace						
Very good	5	1.4	2	0.7	7	1.1
Good	110	29.7	76	26.6	186	28.4
Fair	162	43.8	116	40.5	278	42.4
Poor	66	17.8	60	21.0	126	19.2
Very poor	26	7.0	32	11.2	58	8.8
Indifferent	1	0.3	0	0.0	1	0.1
Job stability during the pandemic						
Very good	2	0.5	2	0.7	4	0.6
Good	47	12.7	31	10.8	78	11.9
Fair	242	65.4	193	67.5	435	66.3
Poor	71	19.2	53	18.5	124	18.9
Very poor	7	1.9	6	2.1	13	2.0
Do not know	1	0.3	1	0.3	2	0.3
Work authorization provided emotional support						
Yes	361	97.6	281	98.2	642	97.9
No	9	2.4	5	1.7	14	2.1
Use of chemical substances in their workplace surroundings (n = 654)						
Yes	22	6.0	26	9.5	48	7.3
No	347	94.0	259	90.5	606	92.7
Working hours a day						
4-8	79	21.4	58	20.2	137	20.9
9-10	154	41.6	138	48.3	292	44.5
> 10	137	37.0	90	31.5	227	34.6
Occupational experience in years						
< 5	3	0.8	2	0.7	5	0.8
5-10	35	9.5	36	12.6	71	10.8
11-20	87	23.5	106	37.1	193	29.4
21-30	108	29.2	73	25.5	181	27.6
> 30	137	37.0	69	24.1	206	31.4
Noise is so loud that one has to shout to communicate (n = 653)						
Yes	250	67.6	213	74.5	463	70.9
No	120	32.4	70	25.5	190	29.1

ELCSA = Latin American and Caribbean Food Security Scale (Escala Latinoamericana y Caribeña de Seguridad Alimentaria).

Overall, 11.1% of respondents reported the use of chemical substances, either at their workplace (3,8%) or in its surrounding areas (7.3%). A total of 61.6% of participants considered the air quality of their workplace to be fair or poor, and 70.9% perceived the noise to be so loud that they had to shout to communicate, an issue more frequently reported by women (Table 2).

### FAMILY DYSFUNCTION AND FOOD INSECURITY

Severe family dysfunction was present in 26.1% of participants, and moderate dysfunction in 20.9%, with severe dysfunction being more common in women’s households (Table 1). Moderate/severe food insecurity during the pandemic affected 44.1% of the sample, with a prevalence of 38.4% among men and 51.4% among women (Table 2).

### MODERATE/SEVERE DEPRESSIVE SYMPTOMS

In the surveyed population, the prevalence of moderate/severe depressive symptoms (MSDSs) was 4.35%, with similar rates in men and women (Table 1).

### SOCIODEMOGRAPHIC CONDITIONS AND HABITS ASSOCIATED WITH DEPRESSIVE SYMPTOMS

Significant associations (p < 0.05) were found between higher prevalence of MSDSs and several worker’s characteristics, such as family type, area of origin, and alcohol consumption. Specifically, individuals aged 30-44 years had a 2.22 times higher prevalence of MSDSs compared to those over 60 years of age (PR = 3.22; 95%CI 1.25-8.27); single-parent households had a 6.54 times greater prevalence than nuclear or extended families (PR = 7.54; 95%CI 2.01-28.31); and urban residents had a 1.83 greater prevalence than rural residents (PR = 2.83; 95%CI 1.16-6.92). Finally, the prevalence of MSDSs was 1.10 times higher among those who consumed alcohol (PR = 2.10; 95%CI1.00-4.45) (Table 3).

**Table 3:** Sociodemographic conditions and habits associated with depressive symptoms in the surveyed working population, Medellín, 2021 (n = 656)

Variable Sociodemographic conditions	Depressive symptoms		Total	Chi-square test (p-value)*	PR (95%CI)
	Moderate/severe n (%)	Absent/mild n (%)			
Biological sex at birth					
Male	16 (4.3)	354 (95.7)	370	0.00 (0.935)	1.03 (0.49-2.14)
Female	12 (4.2)	274 (95.8)	286		1.0
Age in completed years					
18-29	1 (4.1)	23 (95.9)	24	8.31 (0.040)	1.46 (0.18-11.66)
30-44	13 (9.8)	129 (90.2)	132		3.22 (1.25-8.27)
45-59	11 (3.9)	271 (96.1)	282		1.37 (0.52-3.65)
≥ 60	6 (2.8)	205 (97.2)	211		1.0
Head of household					
Yes	27(4.8)	531 (95.2)	558	2.07 (0.150)	4.69 (0.64-34.14)
No	1 (1.0)	96 (99.0)	97		1.0
Marital status					
Without a partner	12 (3.2)	358 (96.8)	370	2.18 (0.139)	0.58 (0.28-1.21)
With a partner	16 (5.6)	270 (94.4)	286		1.0
Educational level in years					
0-5	14 (3.5)	384 (96.5)	398	2.20 (0.332)	0.54 (0.24-1.23)
6-10	5 (4.2)	114 (95.8)	119		0.65 (0.22-1.88)
> 10	9 (6.5)	130 (93.5)	139		1.0
Monthly income in Colombian pesos					
≤ 300.000	2 (1.6)	172 (98.4)	178	7.51 (0.111)	0.13 (0.02-0.77)
301.000-500.000	5 (3.8)	128 (96.2)	133		0.44 (0.11-1.75)
501.000-800.000	12 (5.9)	190 (94.1)	202		0.69 (0.21-2.33)
801.000-1.000.000	6 (5.4)	106 (94.6)	112		0.63 (0.21-2.37)
> 1.000.000	3 (8.6)	32 (91.4)	35		1.0
Household socioeconomic stratum (n = 653)					
Zero or very low	6 (3.1)	186 (96.9)	192	9.19 (0.026)	0.21 (0.06-0.70)
Low	14 (5.0)	269 (95.0)	283		0.33 (0.12-0.94)
Lower-middle	4 (2.6)	147 (97.4)	151		0.18 (0.05-0.67)
Middle	4 (11.5)	23 (88.5)	26		1.00
Family type					
Nuclear or extended	12 (3.3)	349 (96.7)	360	11.57 (0.020)	1.0
Blended	8 (6.5)	116 (93.5)	124		1.95 (0.81-4.65)
One-person	3 (2.7)	107 (97.3)	110		0.82 (0.24-2.86)
Single-parent	2 (25.0)	6 (75.0)	8		7.54 (2.01-28.31)
Restructured or other	3 (5.8)	49 (94.2)	52		1.74 (0.51-5.96)
Cohabitation with other families					
No	21 (3.8)	536 (96.2)	557	2.24 (0.134)	0.53 (0.23-1.22)
Yes	7 (7.1)	92 (92.9	99		1.0
Area de residence with the municipality of origin (n = 651)					
Urban	21 (5.8)	40 (94.2)	360	5.76 (0.016)	2.83 (1.16-6.92)
Rural	6 (2.1)	285 (97.9)	291		1.0
Alcohol consumption					
Yes	10 (7.3)	127 (92.7)	137	3.87 (0.049)	2.10 (1.00-4.45)
No	18 (3.5)	500 (96.5)	518		1.0
Smoking					
Yes	7 (5.6)	118 (94.4)	125	0.66 (0.416)	1.41 (0.61-3.25)
No	21 (4.0)	509 (96.0)	530		1.0

PR = prevalence ratio.

* Statistically significant association when p < 0.05.

Although not statistically significant, a higher prevalence of MSDSs was observed among workers aged 18-29 years and 46-59 years; those from blended, restructured, or other family types; and those who smoked (Table 3).

Conversely, an 87% lower prevalence of MSDSs (p < 0.05) was found among individuals with a monthly income of 500,000 Colombian pesos (COP) or less. Finally, although not statistically significant, a lower prevalence of these symptoms was noted among those earning between 501,000 and 800,000 COP and between 801,000 and 1,000,000 COP at the time of data collection (Table 3).

### WORKING CONDITIONS ASSOCIATED WITH DEPRESSIVE SYMPTOMS

A significant association (p < 0.05) was found between MSDSs and some working conditions: the prevalence of these symptoms was higher among individuals without permission to work (PR = 2.18; 95%CI 1.00-4.74) and lower among those who perceived their job stability as fair (PR = 0.18; 95%CI 0.04-0.85). No significant associations were observed between other working conditions and prevalence of MSDSs; however, these symptoms were more common among individuals who rated their job stability as poor or very poor job before the pandemic and as fair, poor, or very poor during the pandemic, and were less common among those with > 30 years of occupational experience (Table 4).

**Table 4:** Working conditions associated with depressive symptoms in the working population of Medellín, 2021 (n = 656)

Variable	Depressive symptoms		Total	Chi-squared test (p-value)*	PR (95CI)
	Moderate-severe n (%)	Absent-mild n (%)			
Workplace environmental conditions					
Noise is so loud in workplace surroundings that one has to shout to communicate (n = 653)					
Yes	19 (4.1)	444 (95.9)	463	0.00 (0.950)	0.97 (0.43-2.19)
No	8 (4.2)	182 (95.8)	190		1.0
Uses chemical substances at their workplace					
Yes	1 (4.0)	24 (96.0)	25	0.19 (0.662)	0.93 (0.13-6.66)
No	27 (4.3)	604 (95.7)	631		1.0
Use of chemical substances in workplace surroundings (n = 654)					
Yes	2 (4.2)	46 (95.8)	48	0.11 (0.742)	0.97 (0.25-3.80)
No	26 (4.3)	580 (95.7)	606		1.0
Rating of noise at their workplace					
Very tolerable	3 (6.3)	45 (93.7)	48	8.86 (0.115)	1.0
Moderately tolerable	11(3.5)	305 (96.5)	316		0.56 (0.16-1.92)
Indifferent	1 (5.3)	18 (94.7)	19		0.84 (0.09-7.60)
Little tolerable	1 (33.3)	2 (66.7)	3		5.33 (0.77-37.10)
Intolerable	4 (7.7)	48 (92.3)	52		1.23 (0.30-5.22)
Muy intolerable	8 (3.7)	210 (96.3)	218		0.59 (0.16-2.13)
Working conditions					
Work authorization					
No	19 (5.9)	304 (94.1)	323	4.06 (0.044)	2.18 (1.00-4.74)
Yes	9 (2.7)	324 (97.3)	333		1.0
Number of workdays a week					
1-6	13 (3.4)	373 (96.6)	386	1.86 (0.173)	0.61 (0.29-1.25)
7	15 (5.6)	255 (94.4)	270		1.0
Working hours a day					
4-8	8 (5.8)	129 (94.2)	137	3.07 (0.216)	1.0
9-10	8 (2.7)	284 (97.3)	292		0.47 (0.18-1.22)
> 10	12 (5.3)	215 (94.7)	227		0.91 (0.38-2.16)
Occupational experience in years					
≤ 10	6 (7.9)	70 (92.1)	76	6.20 (0.102)	1.0
11-20	11 (5.7)	182 (94.3)	193		0.72 (0.28-1.88)
21-30	7 (3.9)	174 (96.1)	181		0.49 (0.17-1.41)
> 30	4 (2.0)	202 (98.0)	206		0.24 (0.07-0.85)
Job stability before the pandemic (n = 655)					
Very good	8 (8.6)	85 (91.4)	93	7.89 (0.048)	1.0
Good	17 (4.0)	411 (96.0)	428		0.46 (0.21-1.04)
Fair	2 (1.6)	124 (98.4)	126		0.18 (0.04-0.85)
Poor or very poor	1 (12.5)	7 (87.5)	8		1.45 (0.21-10.21)
Job stability during the pandemic (n = 654)					
Very good	2 (2.4)	80 (97.6)	82	1.51 (0.470)	1.0
Fair	18 (4.1)	417 (95.9)	435		1.70 (0.40-7.17)
Poor or very poor	8 (5.8)	129 (94.2)	137		2.39 (0.52-11.0)

PR = prevalence ratio.

* Statistically significant association at p < 0.05.

### CONDITIONS REGARDING MANDATORY ISOLATION, FOOD INSECURITY, AND FAMILY DYSFUNCTION ASSOCIATED WITH DEPRESSIVE SYMPTOMS

A higher prevalence of MSDSs was significantly associated (p < 0.05) with not receiving financial support during mandatory isolation (PR = 4.27; 95%CI 1.16-13.80) and with receiving financial support through their labor association (PR = 8.67; 95%CI 2.35-32.07). Despite lacking statistical significance, a higher prevalence of MSDSs was observed among workers who spent quarantine with peer vendors, relatives, or friends at another household; those who received support from a public or private entity; those who received financial support directly from their labor association; and those experiencing household moderate/severe food insecurity (Table 5).

**Table 5:** Conditions regarding mandatory isolation, food insecurity, and family disfunction associated with depressive symptoms in the surveyed working population, Medellín, Colombia, 2021 (n = 656)

Variable	Depressive symptoms		Total	Chi-square test (p-value)*	RP (IC95%)
	Moderate-severe n (%)	Absent-mild n (%)			
Mandatory isolation conditions					
Self-isolated during the quarantine					
Yes	27 (4.3)	600 (95.7)	627	0.06 (0.805)	1.25 (0.18-8.87)
No	1 (3.4)	28 (96.6)	29		1.0
Duration of mandatory isolation in weeks (n = 628)					
≤ 4	4 (7.0)	53 (93.0)	57	1.78 (0.619)	1.0
5-8	2 (5.0)	38 (95.0)	40		0.71 (0.14-3.70)
9-12	4 (5.6)	67 (94.4)	71		0.80 (0.21-3.07)
> 12	17 (3.7)	443 (96.3)	460		0.53 (0.18-1.51)
Whom they lived with during the quarantine					
Family at their household	20 (3.8)	509 (96.2)	529	6.22 (0.101)	1.0
Relatives or friends at another household	6 (9.2)	65 (90.8)	70		2.23 (0.93-5.38)
Peer vendors	1 (16.7)	5 (83.3)	6		4.41 (0.70-27.76)
Other	1 (2.0)	49 (98.0)	50		0.53 (0.07-3.86)
Received support during quarantine (n = 652)					
No	4 (2.7)	146(97.3)	150	0.79 (0.373)	0.56 (0.20-1.58)
Yes	24 (4.8)	478(95.2)	502		1.0
Received financial assistance during quarantine					
No	21 (6.6)	296 (93.4)	317	5.58 (0.018)	4.17 (1.26-13.80)
Yes	3 (1.6)	186 (98.4)	189		1.0
Is a member of a community organization					
No	2 (2.3)	85 (97.7)	87	0.48 (0.490)	0.50 (0.12-2.08)
Yes	26 (4.6)	543 (95.4)	569		1.0
Received support or collaboration from peer vendors when needed					
Never	11 (5.9)	176 (94.1)	187	5.48 (0.241)	1.0
Almost never	1 (5.6)	17 (94.4)	18		0.95 (0.39-2.29)
Sometimes	2 (1.5)	131 (98.5)	133		0.90 (0.12-6.75)
Almost always	6 (3.2)	183 (96.8)	189		0.24 (0.05-1.12)
Always	8 (6.2)	121 (93.8)	129		0.51 (0.18-1.44)
Peer vendors can be a source of support in times of hardship and crisis					
No	13 (4.2)	298 (95.8)	311	0.01 (0.944)	1.03 (0.49-2.15)
Yes	14 (4.1)	330 (95.9)	344		1.0
Membership in a labor association offered social support					
No	3 (2.9)	99 (97.1)	102	0.21 (0.649)	0.65 (0.20-2.12)
Yes	25 (4.5)	529 (95.5)	554		1.0
Received support from peer vendors during the pandemic (n = 654)					
No	23 (3.7)	600 (96.3)	623	11.15 (0.000)	0.23(0.09-0.56)
Yes	5 (16.1)	26 (83.9)	31		1.0
Received support from their labor association during the pandemic (n = 655)					
No	15 (4.8)	298 (95.2)	313	0.39 (0.531)	1.26 (0.61-2.61)
Yes	13 (3.8)	329 (96.2)	342		1.0
Type of support received from their labor association (n = 338)					
Financial	2 (28.6)	5 (71.4)	7	6.04 (0.013)	8.67 (2.35-32.07)
Food assistance	11 (3.3)	323 (96.7)	334		1.0
Received support from a public or private entity					
No	16 (5.8)	261 (94.2)	277	2.60 (0.107)	1.81 (0.87-3.76)
Yes	12 (3.2)	364 (96.8)	376		1.0
Type of support received from the private sector (n = 377)					
Financial	1 (10.0)	9 (90.0)	10	0.11 (0.740)	3.34 (0.48-23.41)
Food assistance	11 (3.0)	356 (97.0)	367		1.0
Food insecurity and family dysfunction					
Food insecurity					
Moderate/severe	15 (5.2)	274 (94.8)	289	1.07 (0.299)	1.47 (0.71-3.03)
Absent/mild	13 (3.5)	354 (96.5)	367		1.0
Family dysfunction					
Severe	9 (5.3)	162 (94.7)	171	1.37 (0.702)	1.09 (0.45-2.63)
Moderate	5 (3.6)	132 (96.4)	137		0.76 (0.26-2.17)
Mild	4 (2.9)	136 (97.1)	140		0.59 (0.19-1.86)
Absent	10 (4.8)	198 (95.2)	208		1.0

PR = prevalence ratio.

* Statistically significant association at p < 0.05.

### CONDITIONS THAT CONTRIBUTED TO THE OCCURRENCE OF MSDSS IN THE SURVEYED WORKING POPULATION

After adjusting the prevalence of MSDSs for working, workplace environmental, and socioeconomic conditions that were associated (p < 0.05) or had a p-value < 0.10 in bivariate analyses, in order to identify which factors helped explain a higher prevalence of MSDSs, a significant effect (p < 0.05) was found in individuals aged 30-44 years, who showed a 5.99-fold higher prevalence of MSDSs compared to those aged 60 years or older (adjusted PR = 6.99; 95%CI 1.71-28.56). The prevalence of these symptoms was also 2.12 higher among individuals from single-parent or blended families (adjusted PR = 3.12; 95%CI 1.16-8.41), and 8.92 higher among those from nuclear or extended families (adjusted PR = 9.92; 95%CI 1.50-65.66); and 1.35 higher among those who consumed alcohol (adjusted PR = 2.35; 95%CI 00-5.58). A lack of financial support from a public or private entity during mandatory quarantine (adjusted PR = 4.60; 95%CI 1.27-16.64) also significantly contributed (p < 0.05) to the higher prevalence of MSDSs (Table 6).

**Table 6:** Working, workplace environmental, and socioeconomic conditions that helped explain moderate/severe depressive symptoms among the working population included in the study (n = 656)

Variable	Crude PR	LT	UT	Adjusted PR	LT	UT
Model 1. Workers’ sociodemographic conditions and habits						
Age (≥ 60 years Cr.)						
18-29	1.46	0.18	11.66	2.98	0.27	32.90
30-44	3.22	1.25	8.27	6.99	1.71	28.56
45-59	1.37	0.52	3.65	3.14	0.82	12.09
Household socioeconomic stratum (Middle. Cr.)						
Very low	0.21	0.06	0.70	0.13	0.03	0.56
Low	0.33	0.12	0.94	0.21	0.06	0.82
Lower-middle	0.18	0.05	0.67	0.12	0.03	0.57
Family type (Nuclear or extended. Cr.)						
Blended	1.95	0.81	4.65	3.12	1.16	8.41
Unipersonal	0.82	0.24	2.86	1.39	0.36	5.47
Single-parent	7.54	2.01	28.31	9.92	1.50	65.66
Restructured/other	1.74	0.51	5.96	1.20	0.28	5.13
Area of residence within the municipality of origin (Rural. Cr.)						
Urbana	2.83	1.16	6.92	2.46	0.92	6.60
Alcohol consumption (No. Cr.)						
Yes	2.10	1.00	4.45	2.35	1.00	5.58
Model 2. Workplace environmental factors						
Work authorization (Yes. Cr.)						
No	2.18	1.00	4.54	1.74	0.74	4.10
Occupational experience (≤ 10 years Cr.)						
11-20	0.72	0.28	1.88	0.86	0.30	2.46
21-30	0.49	0.17	1.41	0.64	0.20	2.10
> 30	0.24	0.07	0.85	0.32	0.08	1.25
Job stability (Very good. Cr.)						
Good	0.46	0.21	1.04	0.45	0.18	1.08
Fair	0.18	0.04	0.85	0.19	0.04	0.92
Poor or very poor	1.45	0.21	10.21	1.12	0.12	10.62
Model 3. Mandatory isolation and social support during the pandemic						
Received support from a public or private entity (Yes. Cr)						
No	1.81	0.87	3.76	4.60	1.27	16.64
Received support from peer vendors (Yes. Cr)						
No	0.23	0.09	0.56	0.24	0.08	0.69
Type of support received from their labor association during mandatory isolation (Food aid. Cr)						
Financial	8.67	2.35	32.07	4.91	0.51	47.31

LT = lower threshold; PR = prevalence ratio; UT = upper threshold.

A non-statistically significant association was observed between prevalence of MSDSs and the following factors: being aged 18-29 years or 45-59, living in a single-person household, living in the urban area, having received financial support from their workers’ association during mandatory isolation, and lacking permission to work (adjusted PR = 1.74) (Table 6).

Conversely, a lower prevalence of MSDSs was significantly associated (p < 0.05) with living in a very poor (adjusted PR = 0.13; IC 0.03-0.56), poor (adjusted PR = 0.21; IC 0.06-0.82), medio-bajo (adjusted PR = 0.12, IC 0.03-0.57), and moderate job stability (adjusted PR = 0.19, IC 0.04-0.92) (Table 6).

## DISCUSSION

Data for the present study were collected during the peak of the COVID-19 pandemic in Colombia (March-June 2021). As a result, conditions experienced both during the mandatory lockdown and outside of it were examined. The results clearly distinguish between data referring specifically to the lockdown and those reflecting the broader pandemic context.

It is important to note that a snowball sampling approach was used, relying on community leaders, representatives, and the workers themselves. While many of the participants have been involved in the knowledge-building process centered on this population in downtown Medellín for over 19 years, the pandemic context led to the inclusion of workers from the city’s outskirts and rural districts (*corregimientos*). Given the challenges of accessing this population during an exceptional situation, neither probabilistic sampling nor a full census was feasible. Instead, the study prioritized the quality of the information collected. While this may limit the study’s statistical generalizability, it enhances its relevance for public and occupational health.

Overall, the sociodemographic and working conditions of this population either remained unchanged or worsened,^7^ especially among women.^11^ This situation reflects a broader global trend in which the pandemic has negatively impacted psychosocial conditions, increasing risks for mental health issues and other non-communicable chronic diseases. In Latin America, informal workers face sociobiological vulnerability due to the lack of labor regulation and limited access to social security, which left them in need during lockdowns and increased their risk of COVID-19 infection.^12^ The International Labor Organization (ILO) has highlighted the importance of addressing these conditions to safeguard workers’ health and well-being.^13^ Notably, the prevalence of MSDSs in the working population included in the present study was 4.8%, considerably lower than the rates reported both before^7^ and during the pandemic.

Of note, no significant association was found between sex and MSDSs. In comparison, previous research by Garzón et al. conducted with the same population prior to the pandemic reported higher symptom rates (15.7% in men and 15.5% in women).^7,11^ Conversely, the prevalence of MSDSs was significantly higher among individuals aged 30-44 years and 45-59 years across both sexes. According to the Pan American Health Organization (PAHO), these figures are lower than the estimated age-specific prevalence rates of depression, which are above 7.5% in women and 5.5% in men aged 55-74 years.^14^ Notably, 31.7% of older adults (≥ 60 years)^15^ showed a lower prevalence of MSDSs, potentially due to factors such as feeling protected during the pandemic, receiving external support, and exhibiting greater resilience than younger individuals.

In the context of the pandemic, women were more likely than men to assume the role of head of household (89.9 vs. 85.6%), However, their working conditions were more negatively affected by mandatory lockdowns.^16^ Moreover, their participation in the informal labor sector has increased significantly, rising from 38% in 2009 to 43% in 2017. However, women’s working conditions are often more precarious. Globally, there is a well-documented trend of labor insecurity affecting women, characterized by greater difficulty covering expenses, longer working hours, more unpaid or involuntary labor, and limited access to training and occupational health and safety information.^17^ These conditions worsened for this working population during the pandemic.^7,11^

Among our study population, 75% of women and 42% of men did not have a partner. Living in a single-parent household was significantly associated with and contributed to a higher prevalence of MSDSs. The literature indicates that living alone increases the risk of developing depressive symptoms, due to reduced access to support networks.^18^ In turn, belonging to a lower socioeconomic stratum was associated with and contributed to explaining a lower prevalence of MSDSs. This may be related to the concern over access to psychosocial, and especially economic, resources that enable better living conditions.^19^ As socioeconomic status increases, so do financial responsibilities and expectations.

An exploratory analysis on mental health in the metropolitan area of Valle de Aburrá during the pandemic reported that 43.9% of individuals were at risk of developing alcohol-related problems.^20^ These findings are consistent with those of the present study, in which alcohol consumption was associated with and contributed to a higher prevalence of MSDSs. According to PAHO,^12^ the use of central nervous system depressants also rose in Latin America during the pandemic.

Regarding working conditions and the work environment, although no statistically significant associations, a higher prevalence of MSDSs was observed among those who lacked work authorization. This issue, already present before the pandemic,^7^ was exacerbated by lockdown measures that prevented people from working. Furthermore, Municipal authorities suspended or revoked work for street vendors in the city for various reasons, disproportionately affecting women.

Nevertheless, reporting fair, poor, or very poor job stability, both before and during the pandemic, was associated with a higher prevalence of MSDSs. Although this prevalence declined noticeably compared to the pre-pandemic period, this could partly be explained by the fact that these workers were prioritized for food and financial aid, due to their status as a labor-vulnerable population. According to the ILO, this status is defined by unsafe and unhealthy working conditions, lack of skills, low productivity, low or irregular income, long working hours, and limited access to information.^21^ Among the workers evaluated in the present study, greater occupational experience was associated with a lower prevalence of MSDSs a result consistent with what was observed before the pandemic.^7,12^ However, these findings are difficult to compare with other populations of informal workers operating on city streets and sidewalks, as evidence on the topic remains scarce in the pandemic context and particularly for these population groups.

### CONDITIONS REGARDING SUPPORT AND MANDATORY ISOLATION

The absence of support during mandatory quarantine was associated and contributed to a higher prevalence of MSDSs. This is a complex situation, as being mandatorily isolated without assistance directly impacts workers’ income, which dropped by 60% globally in the first month of quarantine and by 80% in Latin America and the Caribbean.^22^ Álvarez et al.,^23^ in turn, reported that support programs and non-contributory social assistance were essential for the subsistence of informal workers during the pandemic. Support received was mainly in the form of food and, to a lesser extent, money, mainly provided by the private sector. A higher prevalence of MSDSs was observed among those who received no support. It is likely that those who received assistance faced greater socio-labor vulnerability, and that the challenges affecting this population intensified during the pandemic.^24^ However, the aid provided served as a protective factor that also helped reduce their MSDSs.

## CONCLUSIONS

Although the COVID-19 pandemic increased the risks to physical and mental health for the working population, and mental health was globally and regionally affected by both mandatory lockdowns and job losses, it is particularly noteworthy that, in the working population assessed in the present study, the prevalence of MSDSs significantly decreased, despite a worsening of their socio-environmental and occupational vulnerability, especially among women. One possible explanation for this outcome is that many workers received support, particularly food assistance from the private sector and state subsides, which may have fostered a sense of protection and recognition by society. Furthermore, the situation highlighted the severe preexisting hardships faced by this population, and how targeted actions such as these support programs had a positive impact on their mental health.
